# Dynamic Spheroidization Mechanism and Its Orientation Dependence of Ti-6Al-2Mo-2V-1Fe Alloy during Subtransus Hot Deformation

**DOI:** 10.3390/ma16175752

**Published:** 2023-08-22

**Authors:** Jinyang Ge, Xiaodong Zhan, Chao Li, Xiaoyong Zhang, Kechao Zhou

**Affiliations:** 1 State Key Laboratory of Powder Metallurgy, Central South University, Changsha 410083, China; 203301071@csu.edu.cn (J.G.);; 2 Hunan Goldsky Titanium Industry Technology Co., Ltd., Changde 410007, China

**Keywords:** α + β titanium alloy, hot deformation, spheroidization mechanism, crystallographic orientations

## Abstract

The dynamic spheroidization mechanism and its orientation dependence in Ti-6Al-2Mo-2V-1Fe alloys during subtransus hot deformation were studied in this work. For this purpose, hot compression tests were carried out at temperatures of 780–880 °C, with strain rates of 0.001–0.1 s^−1^. Based on SEM, EBSD and TEM characterization, the results showed that the aspect ratio of the α phase decreased with increasing deformation temperatures and decreasing strain rates. At 880 °C/0.001 s^−1^, the aspect ratio of the α phase was the smallest at 2.05. The proportion of HAGBs decreased with increasing temperatures and strain rates, which was different from the trend of the spheroidization; this indicated that the formation of HAGBs was not necessary for the spheroidization process. Furthermore, the formation of the α/α interface was related to the evolution of dislocations and twin boundaries at high (880 °C) and low temperatures (780 °C), respectively. Moreover, the dependence of lamellar spheroidization on the crystallographic orientation tilt from the compression direction (θ) was clarified: when θ was between 45° and 60°, both the prism <a> slip and basal <a> slip systems were activated together, which was more favorable for spheroidization. This study could provide guidance for titanium alloy process designs and microstructure regulation.

## 1. Introduction

Dual-phase titanium alloys are widely used in the aerospace, navigation and other fields due to its characteristics of low density, high specific strength and excellent plasticity [1,2]. In order to obtain a desirable microstructure and properties, it is necessary for titanium alloys to go through a series of thermo-mechanical processing (TMP) steps at temperatures above or below the β transus temperature [3]. In general, primary hot working in the β phase region for ingots is implemented to eliminate casting defects and refine the grains [4]. Subsequently, a lamellar microstructure is produced for the billet during the cooling process after primary hot working. Although such lamellar microstructures present with moderate strength and good fracture toughness, their plasticity is poor [5]. In contrast, an equiaxed microstructure presents exhibits superior strength, plasticity and toughness [6,7]. Hence, further hot working in the dual-phase region is necessary to break down the lamellar microstructure (also referred to as spheroidization of the lamellar microstructure) and then obtain the equiaxed microstructure. 

It is quite difficult to achieve the spheroidization of a lamellar microstructure for titanium alloys from the viewpoint of workability and microstructure regulation. As a result, a large number of studies have been devoted to clarifying the factors affecting dynamic spheroidization and the mechanisms involved. The dynamic spheroidization of lamellar microstructures significantly determines the initial microstructure and processing conditions during the hot deformation process [8]. For example, Buzolin et al. [9] systematically investigated the effects of deformation parameters on the dynamic spheroidization of the α phase for a Ti-6Al-4V alloy in the dual phase region; they found that the spherical α was more likely to form at lower strain rates and higher temperatures. Wu et al. [10] established the dynamic spheroidization kinetics of a TA15 titanium alloy with a lamellar microstructure at different deformation parameters. Shell et al. [11] revealed that dynamic spheroidization kinetics vary with the α colony size for lamellar microstructures. The spheroidization rate of a fine α colony was faster than that of a coarse α colony. In addition, the mechanism of spheroidization during hot deformation has been explained—the widely accepted mechanism of dynamic spheroidization was previously thought to consist of three steps: the formation of α/α interfaces during deformation, βwedging along the α/α intra-boundaries and boundary splitting [8,12,13,14,15,16]. The α/α intra-boundaries are mainly formed by high-energy defects such as dislocations, twins and shear bands, providing the necessary driving force for the subsequent instability of the α lamellae [17]. Furthermore, this is accompanied by variations in the accumulated misorientation within α lamellae during the dynamic spheroidization process [18]. 

Recently, the relationship between the spheroidization mechanism and deformation parameters—especially the deformation temperature—has been investigated. For instance, Zherebtsov et al. [19] reported the spheroidization mechanism of a Ti-6Al-4V alloy during hot deformation at 600 °C and 800 °C; they concluded that spheroidization was mainly accomplished by dislocation slipping-induced α substructure evolution at 800 °C, while restricted spheroidization appeared under the action of dynamic recrystallization at 600 °C. Chong et al. [20] investigated twinning as a spheroidization mechanism of a Ti-6Al-4V alloy during hot deformation at 700 °C. Besides this, researchers have found that the spheroidization rates in different regions are still different even under identical initial microstructure and deformation conditions; the reason for this was thought to be the dependence of sphericalization on the orientation of the α colony [21,22]. Mironov et al. [23] reported that α lamellae parallel to the loading direction are more easily broken up, presenting a higher spheroidization efficiency during hot processing for a Ti-6Al-4V alloy. Roy et al. [24,25,26] reported that the formation of sub-boundaries within α lamellae and the separation of α lamellae for a Ti-6Al-4V alloy were all crystallographic orientation-dependent. Wang et al. [27] reported that the activation of the basal <a> slip and prism <a> slip promoted the generation of α/α boundaries. Notably, the activation of the slip systems was related to the crystallographic orientation of the α phase with respect to the compression direction. However, there was still an absence of a further explanation concerning the correlation of the spheroidization mechanism with the crystallographic orientation and compression direction.

In this study, isothermal compression tests were carried out to clarify the dynamic spheroidization mechanism and its orientation dependence in a Ti-6Al-2Mo-2V-1Fe titanium alloy. The microstructure evolution under various deformation conditions was investigated. The correlation of spheroidization mechanisms and temperatures has also been revealed in detail. The dependence of spheroidization on the crystallographic orientation of the α relative to the compression axis was also clarified. These results could guide the design of hot working and the microstructure regulation of dual-phase titanium alloys.

## 2. Experimental Procedure

The specific chemical composition of the experimental material in the present work was 6.2 Al, 2.1 Mo, 2 V and 0.9 Fe, balanced with Ti (wt.%). The as-received alloys were supplied in the form of forged bars that were subjected to the ingot breakdown process in the single β region. The β transus temperature was determined by the metallographic method to be 915 ± 5 °C. The initial microstructure was a typical lamellar microstructure. Cylindrical specimens 10 mm in diameter and 15 mm in height were cut from the billet for hot compression experiments. Uniaxial hot compression experiments were carried out at the deformation temperatures of 780 °C, 830 °C and 880 °C with strain rates of 0.001 s^−1^, 0.01 s^−1^ and 0.1 s^−1^ on a Gleeble-3500 compressor. In order to reduce friction, the top and bottom surfaces of the samples were covered with high-temperature lubricant and tantalum sheets were placed on both ends of the sample and the indenter. Samples were heated to a preset temperature at a heating rate of 10 °C/s and held for 5 min to ensure a uniform temperature before compression. Samples were compressed until the true strain was 0.9, followed by immediate water quenching to retain the high temperature deformation microstructure. In order to elucidate the spheroidization process under high and low temperature conditions, two sets of specimens with true strains of 0.2, 0.5 at 780 °C/0.01 s^−1^ and 880 °C/0.01 s^−1^ were also obtained. 

**Figure 1:**
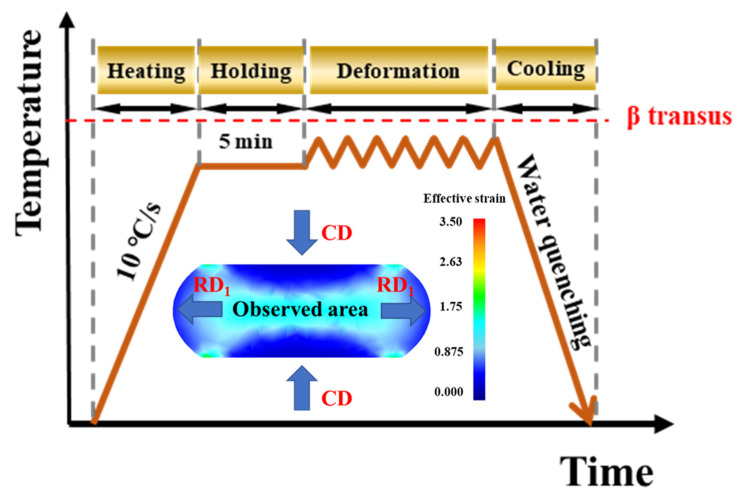
Schedule for the hot compression process and finite element simulation results for the strain region of the deformed specimen.

The finite element simulation results for the strain region of the deformed specimen is also shown in Figure 1. The center of the samples was the main observed and characterized area, where the strain was the required strain value for the experiments. Deformed samples were cut along the compression axis for microstructure observation. The surfaces of the samples were ground, polished, etched with Kroll’s etching solution—10% nitric acid, 30% hydrofluoric acid and 60% water—and then examined on a Tescan Mira4 scanning electron microscope with an accelerating voltage of 15 kV. The aspect ratios and volume fractions of the α phase were statistically calculated by Image J v1.8.0 software. For details such as the grain boundary features and orientation features, a field-emission SEM (Helios Nano Lab G3 UC) equipped with an EBSD detector was employed to characterize the microstructure. The collected data were processed using OIM v8 software. The specimen coordinate system was chosen with the OA_1_// radial directions (RD_1_) and OA2// the compression direction (CD). In order to achieve the surface quality required for the EBSD measurements, the samples were electropolished with a solution of 10% perchloric acid, 30% n-butanol and 60% methanol at −25 °C. In addition, more microscopic features were characterized using a Transmission Electron Microscope (TEM) Tecnai F20 G2 at a voltage of 200 kV. TEM foils were cut from the core of the deformed samples—first mechanically thinned to about 5 μm, and then electropolished using a twin-jet electropolishing device (Denmar Struers A/S).

## 3. Results and Discussion

### 3.1. The Initial Microstructure

**Figure 2:**
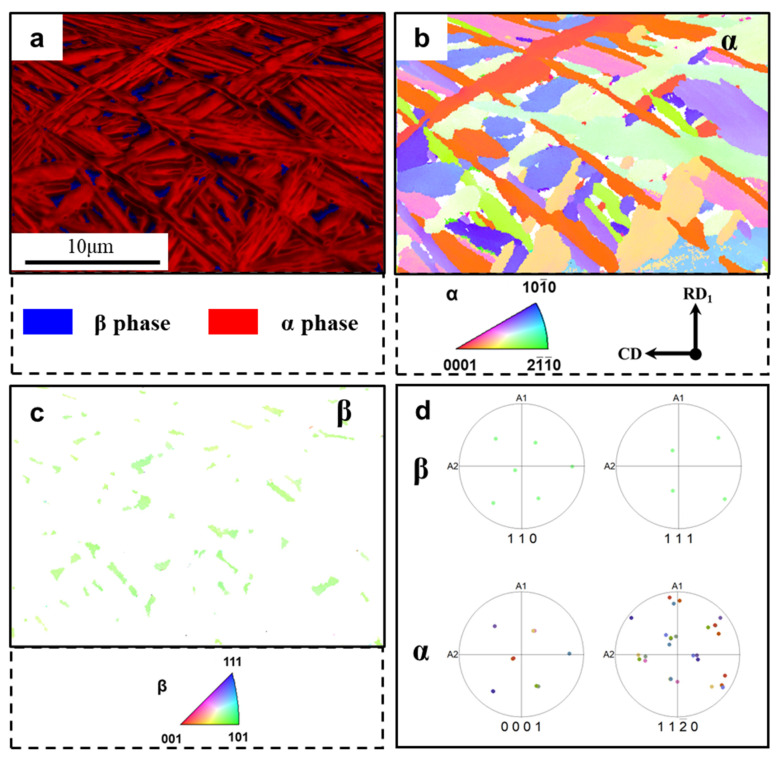
Initial lamellar microstructure of the experiment material. (**a**) Phase distribution (FD) map + image quality map (IQ). Inverse pole figure (IPF) maps of (**b**) α phase and (**c**) β phase. (**d**) Pole figures (PFs) of the lamellar α and β phase.

The initial microstructure information of the as-received alloy is shown in Figure 2. The initial microstructure consisted of lamellar α and a very slight fraction (Vol. 3.6%) of β phase (Figure 2a). Furthermore, the thickness of the α lamellae was about 1 μm. Different colors of α phase represent different α variants (Figure 2b). There was only one orientation of the parent β phase (Figure 2c). According to the pole figures in Figure 2d, it could be confirmed that the α and β phase followed the Burgers orientation relationship: {0001}_α_//{110}_β_ and <11$\overset{—}{2}$0>_α_//<111>_β_. The relationship between the α precipitated phase and the parent β phase has been reported in the lamellar microstructures and transformed microstructures of other titanium alloys [28].

### 3.2. Thermal Plastic Flow Behavior

**Figure 3:**
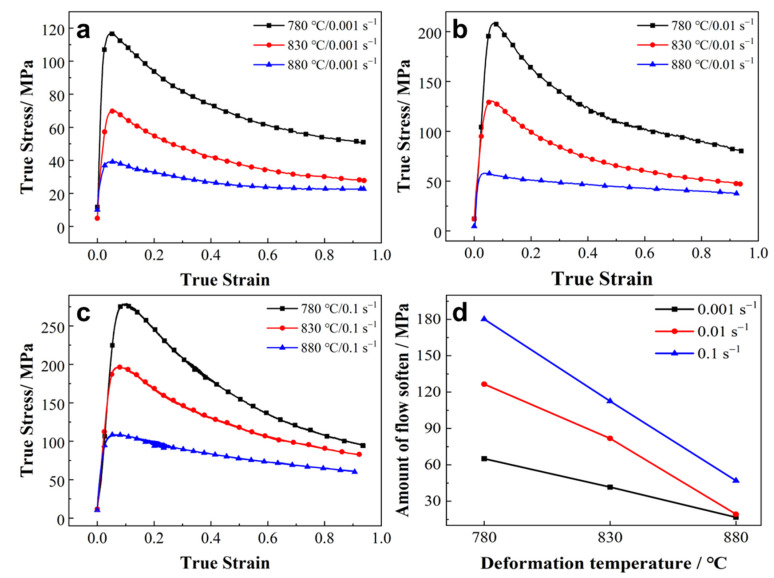
The stress–strain curves of the experimental material with a lamellar microstructure at different temperatures, with strain rates of (**a**) 0.001 s^−1^, (**b**) 0.01 s^−1^ and (**c**) 0.1 s^−1^ and (**d**) the amount of flow softening at different deformation conditions.

Figure 3a–c shows the stress–strain curves of the experimental alloy with a lamellar microstructure under various hot deformation conditions. As can be seen from all of the curves, the true stress reached a peak value at small strains (ε < 0.1), after which, continuous flow softening occurred. Furthermore, the stress–strain curves tended to stabilize until the true strain reached 0.9. The deformation temperature and strain rate significantly affected the characteristics of the stress–strain curves. The peak stress dramatically decreased with increases in the deformation temperature and decreases in the strain rate. The maximum peak stress was 278 MPa at 780 °C, with a strain rate of 0.1 s^−1^. In contrast, the minimum peak stress was only 39 MPa at 880 °C, with a strain rate of 0.001 s^−1^. This trend is consistent with other titanium alloys [29,30]. The amount of flow softening under different deformation conditions is shown in Figure 3d. The trend of the flow softening values is similar to that of the peak stress, decreasing with increases in the deformation temperature and decreases in the strain rate. Previous studies have shown that flow softening could be caused by adiabatic effects and microstructure evolution, such as the spheroidization of α lamellae [31], texture evolution [32] and β recrystallization [12].

### 3.3. Morphological Characteristic of the α Lamellae

**Figure 4:**
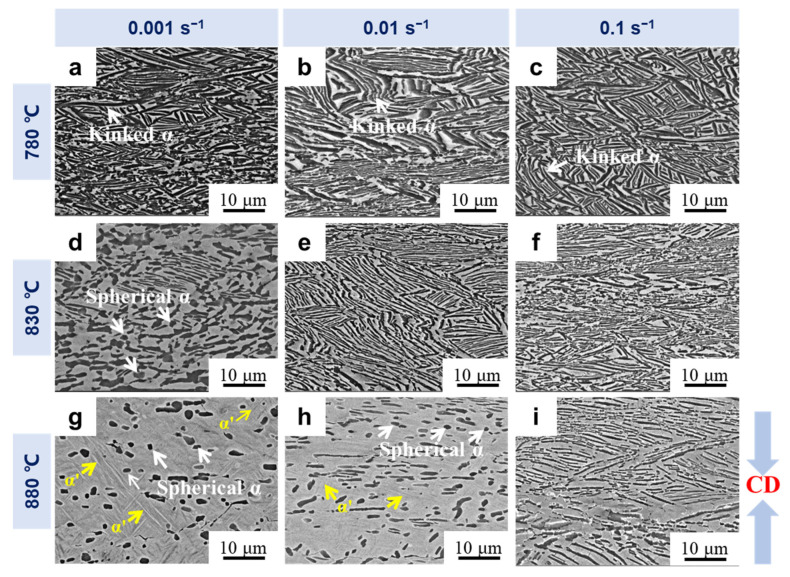
The SEM micrographs (BSE mode) of the deformed microstructures with different deformation conditions. The corresponding deformation temperatures and strain rates are marked on the left and top of the SEM micrographs, respectively. (**a**) 780 °C/0.001 s^−1^; (**b**) 780 °C/0.01 s^−1^; (**c**) 780 °C/0.1 s^−1^; (**d**) 830 °C/0.001 s^−1^; (**e**) 830 °C/0.01 s^−1^; (**f**) 830 °C/0.1 s^−1^; (**g**) 880 °C/0.001 s^−1^; (**h**) 880 °C/0.01 s^−1^; and (**i**) 880 °C/0.1 s^−1^. The white and yellow arrows in the SEM micrographs denoting the α and α’ phases, respectively.

**Figure 5:**
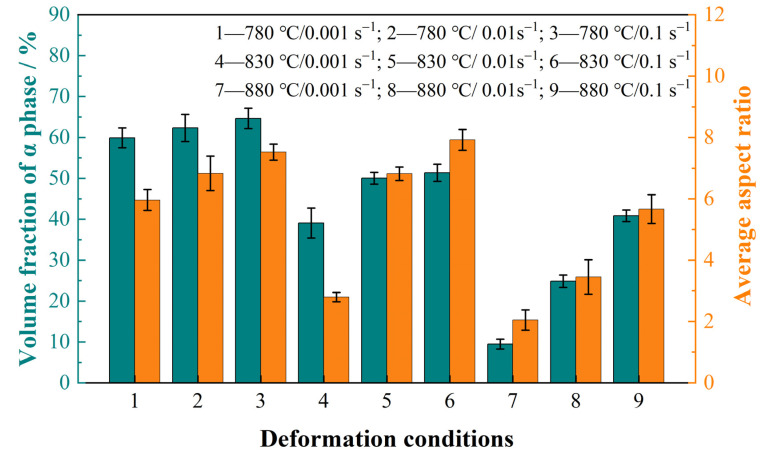
Volume fraction and aspect ratio of the α phase under various deformation conditions.

The α morphology of the experimental material under various hot compression conditions is shown in Figure 4. The lamellar α underwent significant bending and rotation during the deformation in the dual phase region. Furthermore, the spherical α phase was observed under different hot compression conditions. The aspect ratios of the α lamellae increased with increasing strain rates and decreasing deformation temperatures. The corresponding quantitative results for the α aspect ratio are presented in Figure 5. The aspect ratios of the α phase were 2.79, 6.80 and 7.32 at 830 °C, with strain rates of 0.001 s^−1^, 0.01 s^−1^ and 0.1 s^−1^, respectively. The low strain rates allowed for sufficient time to achieve α/α interface separation, and then promoted dynamic spheroidization of the α lamellae. The deformation temperature also affected the aspect ratio of the α phase. When the stain rate was 0.001 s^−1^ and the deformation temperatures increased from 780 °C to 880 °C, the aspect ratio of the α phase decreased from 5.96 to 2.05 (Figure 5). This suggests that higher deformation temperatures are more conducive to dynamic spheroidization of the α lamellae, which is relevant to the fast diffusion rate of solute atoms at high temperatures [17]. Furthermore, the α’ phase was also observed in the samples after deformation at 880 °C/0.001 s^−1^ and 880 °C/0.001 s^−1^. 

The volume fraction of the α phase under different deformation conditions is also shown in Figure 5. Increases in temperature and decreases in strain rates both led to a decrease in the volume fraction of the α phase. In particular, the volume fraction of the α phase was only 9.5% at 880 °C/0.001 s^−1^. Normally, the α phase dissolves under the effect of phase equilibrium at high temperatures. As the strain rate gets lower, the α phase takes longer to dissolve, resulting in a lower volume fraction of the α phase. Besides this, dynamic phase transformation is easily induced at high temperatures and low strain rates [33]. These two reasons together led to the changes in the volume fraction of the α phase under the various deformation conditions.

### 3.4. Relationship between Dynamic Spheroidization and the Dynamic Restoration of α Lamellae

**Figure 6:**
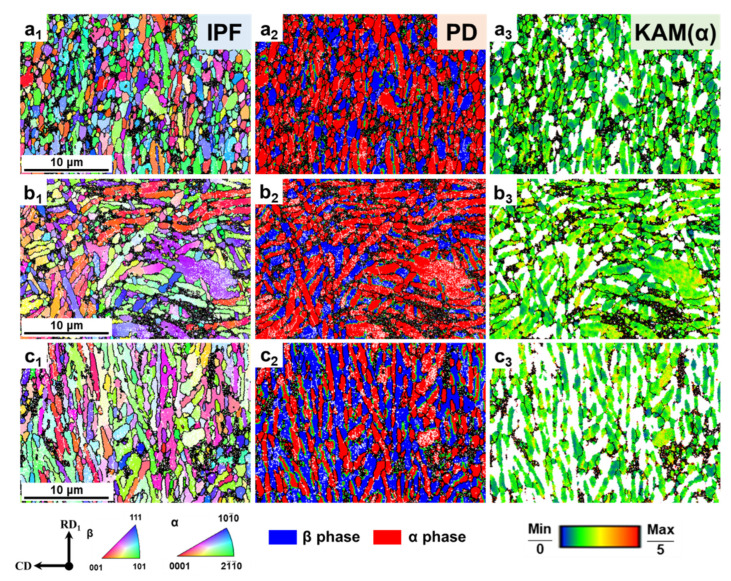
EBSD results of the alloy deformed under the conditions of (**a_1_**–**a_3_**) 780 °C/0.001 s^−1^; (**b_1_**–**b_3_**) 780 °C/0.1 s^−1^ and (**c_1_**–**c_3_**) 880 °C/0.1 s^−1^.

**Figure 7:**
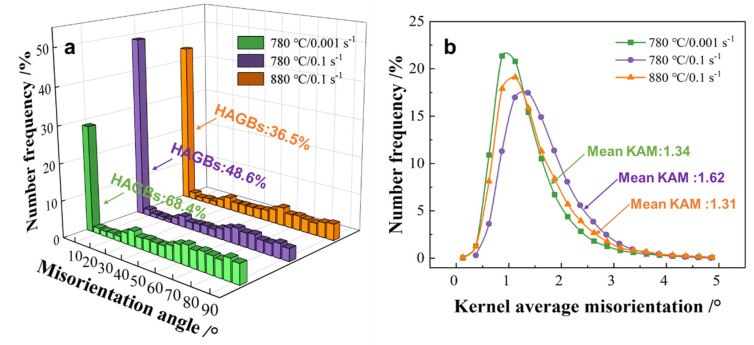
(**a**) Misorientation distributions and (**b**) Kernel average misorientation distributions of α lamellae under different deformation conditions.

The EBSD results of the alloys deformed at 780 °C/0.001 s^−1^, 780 °C/0.1 s^−1^ and 880 °C/0.1 s^−1^ are presented in Figure 6, which contains IPF maps, FD maps and kernel average misorientation (KAM) maps. The high angle boundaries (>15°, HAGBs) and low angle grain boundaries (2~15°, LAGBs) are indicated by the black and white lines in the IPF maps, respectively. The KAM index is often used to represent the local misorientation caused by geometrically necessary dislocations [34]. For the specimen deformed at 780 °C/0.001 s^−1^, the long α lamellae were split into short ones by the HAGBs (Figure 6(a_1_)). The percentage of HAGBs and the average misorientation of the α lamellae reached 68.4% and 43.07°, respectively (Figure 7a)—suggesting that dynamic recrystallization was the main restoration mechanism of the α phase at 780 °C/0.001 s^−1^. Low KAM values were exhibited within the DRXed α grains, while relatively high KAM values were found near the sub-boundaries (Figure 6(a_3_)); this indicates that the occurrence of dynamic recrystallization is effective in reducing the distortion degree within the α phase. When the strain rate increased from 0.001 s^−1^ to 0.1 s^−1^, the percentage of HAGBs and the average misorientation decreased to 48.6% and 31.6°, respectively (Figure 7a). Furthermore, the average KAM value increased from 1.34 to 1.62. These results suggest that dynamic recrystallization is inhibited by increasing strain rates. 

As the deformation temperature increased from 780 °C to 880 °C, the proportion of HAGBs and the average misorientation decreased to 36.5% and 33.9°, respectively (Figure 7a). Furthermore, the average KAM value of 1.31 was lower at 880 °C than that at 780 °C. The above results show that dynamic recrystallization was suppressed as the deformation temperature increased. However, it is already known that dynamic spheroidization is more significant at higher temperature from Figure 4. The dynamic recrystallization and dynamic spheroidization of α lamellae show opposite trends; as a result, the occurrence of dynamic recrystallization is non-essential for the spheroidization of α lamellae.

### 3.5. Dynamic Spheroidization Mechanism of α Lamellae

#### 3.5.1. Correlation of Spheroidization Mechanisms and Deformation Temperature

**Figure 8:**
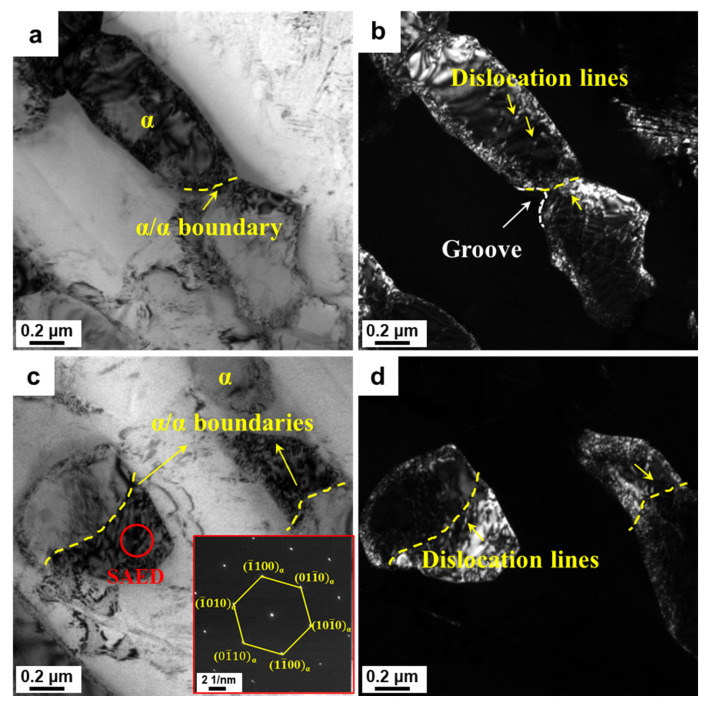
TEM results demonstrating the deformation mechanism based on the dislocation slip of α lamellae under the condition of 880 °C/0.01 s^−1^/60%: (**a**,**c**) bright field (BF) images of α lamellae and corresponding (**b**,**d**) dark field (DF) images, respectively. The red circle representing the position of selected area electron diffraction pattern (SAED).

**Figure 9:**
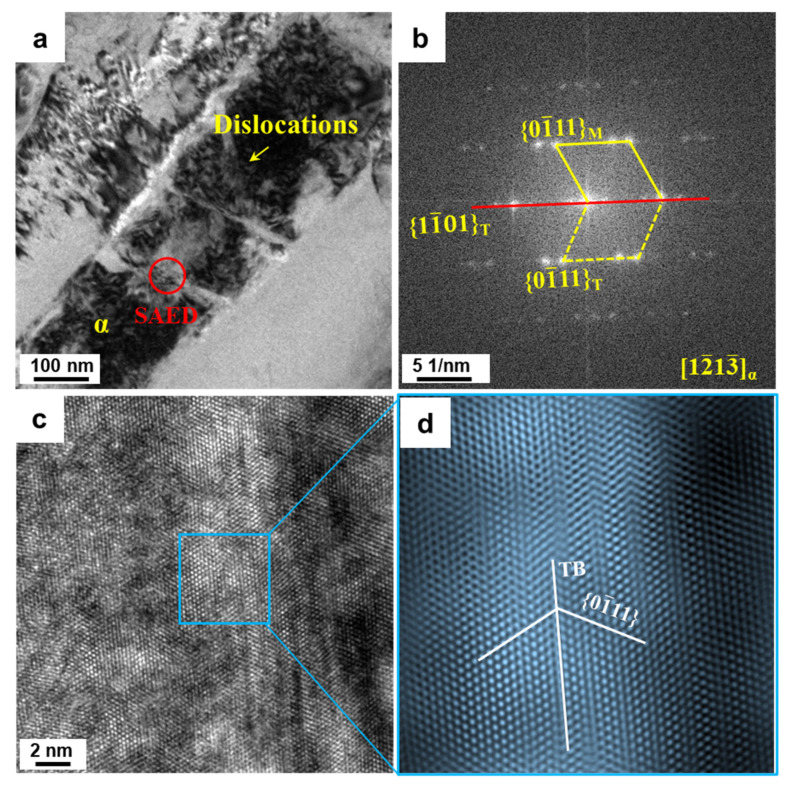
TEM results demonstrating the deformation mechanism based on twinning deformation at 780 °C/0.01s^−1^ with 0.9 strain: (**a**) BF-TEM images, (**b**) SAED pattern along the [1$\bar{2}1\bar{3}$] zone axis corresponding to the red circle of (**a**), (**c**) high-resolution images image of twins and (**d**) inverse fast Fourier-filtered (IFFT) image transformed from blue frame in (**c**).

The formation of the α/α interfaces within the α lamellae is a key step for spheroidization during the hot deformation of titanium alloys. High-energy defects such as dislocations and twins provide the preconditions for the formation of α/α interfaces within lamellae [20,35,36]. The formation of these high-energy defects is closely related to the deformation temperature. In this section, two deformation temperatures of 880 °C and 780 °C were selected to investigate the deformation mechanism of the α lamellae. The deformed microstructures at 880 °C with a strain rate of 0.01 s^−1^ were characterized by TEM (Figure 8). Figure 8a illustrates that the α lamellae were divided into two sub-grains with different orientations. A groove-like microstructural feature appeared at the α/β interface. According to the corresponding dark field (DF) image (Figure 8b), a large number of dislocation and dislocation lines near the grooves were observed. In addition, a sub-boundary is observed across the spherical α phase in Figure 8c,d. Analogously, a large number of dislocations occurred near sub-boundaries within the spherical α phase. It could be speculated that the spherical α phase would probably be broken up should the strain continue to increase. Therefore, the formation of sub-boundaries is mainly dependent on dislocation rearrangement during high-temperature deformation. When the deformation temperature decreased to 780 °C, several parallel or crossed plate-like structures were found across the α lamellae (Figure 9a). Based on the diffraction patterns along the [1$\bar{2}1\bar{3}$] zone axis in selected regions (Figure 9b), the plate-like structures were identified as {10$\bar{1}$1} compression twins. High-resolution TEM images and corresponding inverse fast Fourier-filtered (IFFT) information (Figure 9c,d) further demonstrated the twinning type of the α phase. Furthermore, substantial dislocations piled up at the twin interface. During deformation at a low temperature, the initiation of twinning and the pile-up of dislocations near the twinning interface led to stress concentration, which facilitated the interface separation of the α phase.

**Figure 10:**
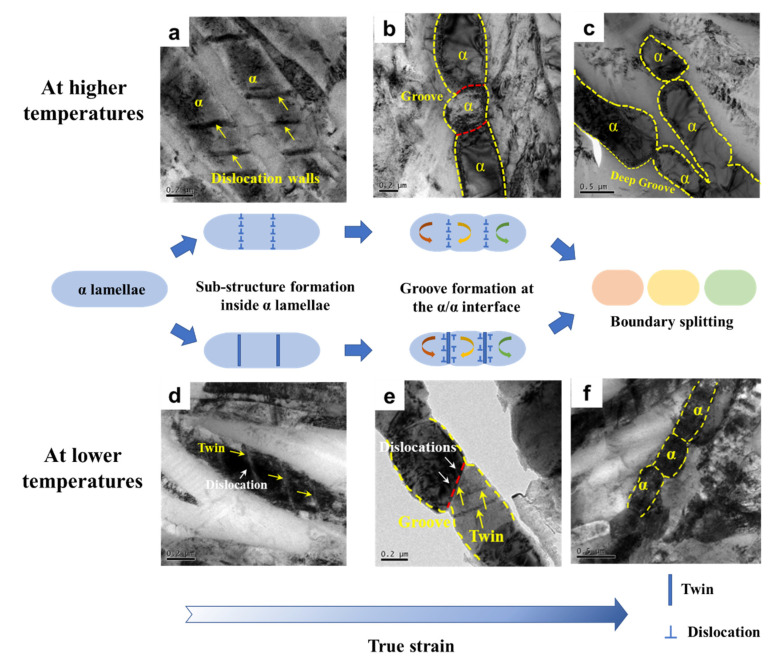
Schematic diagrams demonstrating the dynamic spheroidization mechanism of the α lamellae with the variation of strain at higher and lower temperatures. TEM results showing the dynamic spheroidization process at 880 °C with the true strain of (**a**) 0.2, (**b**) 0.5, (**c**) 0.9 and at 780 °C with the true strain of (**d**) 0.2, (**e**) 0.5, (**f**) 0.9, respectively.

In order to further investigate the process of dynamic spheroidization, specimens compressed to a true strain of 0.2, 0.5 at temperatures of 880 °C and 780 °C and a strain rate of 0.001 s^−1^, respectively, were also obtained. Furthermore, their microstructures were characterized by TEM, as shown in Figure 10. At 880 °C, dislocations and dislocation walls were generated within the α lamellae when the true strain reached 0.2 (Figure 10a). These dislocation walls gradually developed into α/α boundaries with increasing strain up to 0.5. At the same time, the formation of the α/α interface led to the destruction of the original interfacial energy system [37,38]. To reduce the interfacial energy, grooves were formed and the β matrix wedged into the α lamellae along the α/α boundaries (Figure 10b). This process was also accompanied by elemental diffusion, whereby α-type stabilizing elements within the non-equilibrium β matrix migrated into the α phase [39,40,41]. Besides this, the groove depth was also influenced by the misorientation of the α/α boundaries. Therefore, HAGBs were more likely to show development of the groove depth [42]. By further increasing the strain to 0.9, the α lamellae gradually sphered to reduce the total interfacial energy (Figure 10c). At the lower temperatures, the spheroidization mechanism of the α lamellae—on the basis of the formation of sub-interfaces by twins—can be described as follows: Firstly, twinning occurred within the α lamellae when the true strain reached 0.2 (Figure 10d). As the strain increased to 0.5, more slip systems were activated, leading to dislocations becoming enriched in the vicinity of the twin boundaries. The α phase started to fracture along the twin under the action of external forces, and the twins were transformed into an α/α interface (Figure 10e). As the strain continued to increase to 0.9, the β matrix penetrated the α phase along the α/α boundaries. Furthermore, the α lamellae were gradually broken up (Figure 10f). Due to the decreased diffusion rate of the solute atomic at low temperatures, the curvature of the grooves will be smaller with the same strain at low temperatures. The corresponding schematic diagram of the spheroidization mechanism is also displayed in Figure 10. The fragmentation and spheroidization of the α lamellae in titanium alloys involves three steps: (i) the formation of α/α boundaries by dislocations and twin boundaries, (ii) the wedging of the β matrix along the α/α boundaries and (iii) the separation and spheroidization of the α phase.

#### 3.5.2. Dependence of Spheroidization on Lamellar Orientation

The spheroidization behavior of α lamellae also depends on the orientation characteristics of the α lamellae in addition to the external deformation conditions [17,43,44]. From Section 3.5.1, it is known that dislocation slipping is the main deformation mechanism of the α phase under high-temperature conditions. A favorable orientation for α lamellae promotes the activation of multiple slip systems, thereby facilitating the process of spheroidization. In contrast, spheroidization will be delayed by the limited crystal rotation for α lamellae with poor orientations. Nevertheless, the activation of the slip systems is also related to the direction of the loading direction. Therefore, it is essential to analyze the initiation of the dislocation slip system based on the orientation of the α lamellae and the loading direction. Additionally, its effects on spheroidization behavior will be further elucidated.

**Figure 11:**
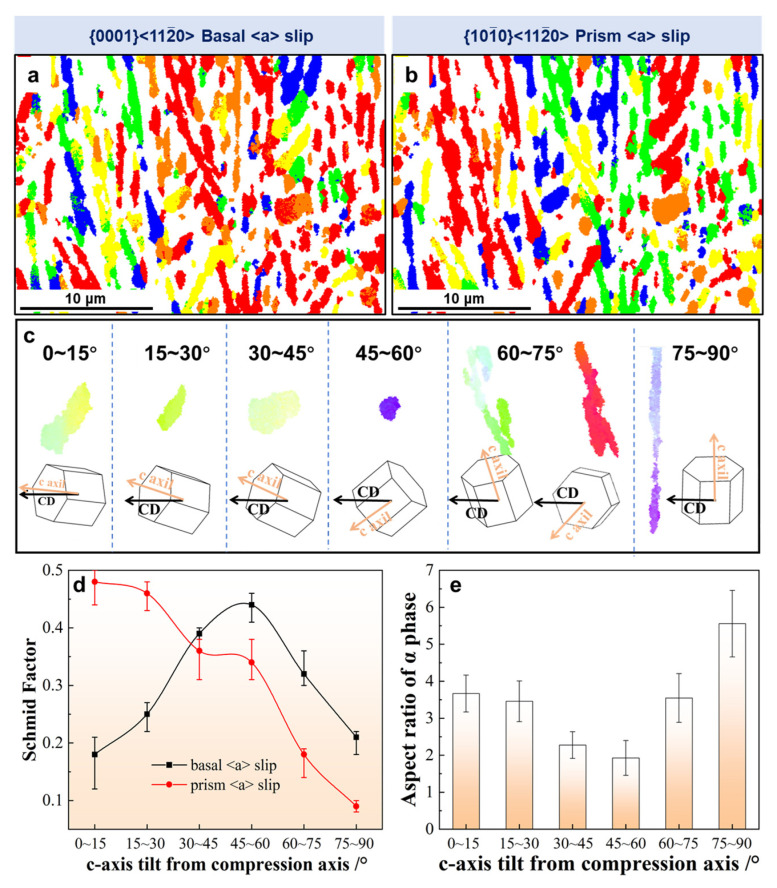
Schmid factor distribution maps of the (**a**) basal <a> slip and (**b**) prism <a> slip of α phase for the specimen deformed at 880 °C/0.1 s^−1^. (**c**) Typical α was selected from (**a**) according to the alignment of the c-axis with respect to the compression direction. Relationship between the (**d**) Schmid factors of the basal <a> slip and prism <a> slip systems as well as the (**e**) aspect ratio and c-axis tilt from the compression direction.

Schmid factor distribution maps of the basal <a> slip and prism <a> slip of the α phase for the specimen during deformation at 880 °C/0.1 s^−1^ are shown in Figure 11a and b, respectively. The activation of the slip systems is closely related to the c-axis tilt towards the compression direction. The angle between the c-axis of the α lamellae and the compression direction is defined as θ. In order to facilitate understanding of the activation of slip systems in α lamellae with different orientations, typical α were selected according to the alignment of the c-axis with respect to the compression direction (Figure 11c). Furthermore, the variation in the Schmid factors of the basal <a> slip and prism <a> slip systems with a c-axis tilt from the compression direction is summarized in Figure 11d. When θ was around 45°, both the prism <a> slip and basal <a> slip of the α phase is activated, providing favorable conditions for the formation of the α/α interface within the lamellae. When the c-axis was close to parallel or perpendicular to the compression direction, the basal <a> slip is hardly likely to be activated. The α phase had two orientations of <0001> and <11$\bar{2}$0> when the θ was 75–90° (Figure 11c). However, the prism <a> slip systems of the α phase with the two orientations were difficult to activate. An α phase with an unfavorable orientation may rotate around the c-axis, bringing into it a more favorable orientation to initiate basal/prism slip; this was confirmed by the presence of low levels of misorientation in the lamellae when θ was 0–15° or 75–90°, according to the IPF color (Figure 11c). Wang et al. [27] investigated whether deformation resistance would reach its lowest value when θ was around 50°—a case in which both the basal <a> plus prism <a> slip systems of the α phase could be activated. The results were similar to those found in the present study. The relationship between the aspect ratio of the α phase and the c-axis tilt from the compression direction during deformation at 880 °C and 0.01 s^−1^ is presented in Figure 11e. The aspect ratio of the α phase decreased with θ, reaching a minimum value at 45–60° and then increasing. As a result, the spheroidization of α lamellae is more favorable when both the prism <a> slip and basal <a> slip systems are activated together.

As mentioned above, the spheroidization behavior differs when the c-axis of the α lamellae tilt from the compression axis varies, which can lead to α spheroidization inhomogeneity. In recent years, researchers have proposed that spheroidization inhomogeneity can be improved by changing the strain path [43,45].

## 4. Conclusions

In the present study, the dynamic spheroidization of a Ti-6Al-2Mo-2V-1Fe alloy during subtransus hot deformation, its mechanism and its orientation dependence were investigated. By detailed microstructure characterization and quantitative analysis, the following conclusions can be drawn:

(1) The peak stress and the amount of flow softening decreased with increases in deformation temperature and decreases in strain rates. At 880 °C/0.001 s^−1^, the aspect ratio of the α phase was the smallest, at 2.05.
(2) The proportion of HAGBs decreased with increasing temperatures and strain rates, which was different from the trend of the spheroidization. Furthermore, the formation of HAGBs was not necessary for the spheroidization process.
(3) The spheroidization mechanisms of α lamellae under high and low-temperature deformation conditions were revealed, comprised of three steps: (i) the formation of sub-interfaces by dislocation slipping at 880 °C and by twinning at 780 °C, respectively, (ii) wedging of the β matrix along the α/α sub-interfaces and (iii) separation and spheroidization of the α phase.
(4) The dependence of lamellar spheroidization on the crystallographic orientation tilt from the compression direction (θ) was clarified. When θ was between 45 and 60°, both the prism <a> slip and basal <a> slip systems were activated together, which was more favorable for spheroidization.

## Data Availability

Not applicable.
